# Supplementary material for: Tailoring of Novel Azithromycin-Loaded Zinc Oxide Nanoparticles for Wound Healing
Source: Pharmaceutics. 2022 Jan 5;14(1):111. doi: 10.3390/pharmaceutics14010111 (PMC8780377; doi:10.3390/pharmaceutics14010111)
Supplement: Supplementary file 1 [file pharmaceutics-14-00111-s001.zip › pharmaceutics-1514182-supplementary.pdf]

pharmaceutics

MDPI

Supplementary Materials: Tailoring of Novel Azithromycin-Loaded Zinc Oxid Nanoparticles for Wound Healing

Mohammed S. Saddik, Mahmoud M.A. Elsayed *, Mohamed A. El-Mokhtar, Haitham Sedky, Jelan A. Abdel-Aleem, Ahmed M. Abu-Dief, Mostafa F. Al-Hakkani, Hazem L. Hussein, Samah A. Al-Shelkamy, Fatma Y. Meligy, Ali Khames and Heba A. Abou-Taleb

Table S1. X – ray data of the prepared ZnO nanoparticles.

2θ° Reference	2θ°hkl Measured	Miller indices		
		h	k	ℓ
31.7729	31.775	1	0	0
34.4268	34.425	0	0	2
36.2585	36.275	1	0	1
47.5466	47.525	1	0	2
56.6023	56.575	1	1	0
62.8681	62.875	1	0	3
66.3862	66.375	2	0	0
67.9599	67.925	1	1	2
69.0953	69.075	2	0	1
72.5819	72.525	0	0	4
76.9756	76.975	2	0	2
Average	---	---	---	---
